# Di­chlorido­bis­(3-{[(pyridin-2-yl)methyl­idene]amino}­benzoic acid-κ^2^*N*,*N*′)cobalt(II) methanol monosolvate monohydrate

**DOI:** 10.1107/S2414314626002154

**Published:** 2026-03-03

**Authors:** Olga Yu. Vassilyeva, Vladimir N. Kokozay, Brian W. Skelton

**Affiliations:** a Department of Chemistry, Taras Shevchenko National University of Kyiv, 12 Hetman Pavlo Skoropadskyi St., Kyiv 01033, Ukraine; b School of Molecular Sciences, M310, University of Western Australia, Perth, WA 6009, Australia

**Keywords:** crystal structure, cobalt(II) complex, Schiff base ligand

## Abstract

The ortho­rhom­bic (space group *Pna*2_1_) unit cell of the title compound, [CoCl_2_(C_13_H_10_N_2_O_2_)_2_]·CH_3_OH·H_2_O, contains eight complex mol­ecules, four *A* and four *B*, along with sixteen solvent mol­ecules of crystallization. The two independent Co^II^ atoms are coordinated by imine and pyridine N atoms from each of two neutral pyridinyl­imino­benzoic acids and two *cis*-chlorido ligands in a distorted octa­hedral coordination environment. In the crystal, the complex mol­ecules pack loosely with the closest Co⋯Co separation exceeding 7.7 Å. The uncoordinated carb­oxy­lic groups on the ligands are presumably hydrogen-bonded to the solvent methanol and water mol­ecules.

## Abstract 2

Two chlorido and two chelating pyridinyl­imino­benzoic acid ligands coordinate a cobalt(II) centre with the carb­oxy­lic being non-coordinating.

## Graphical abstract

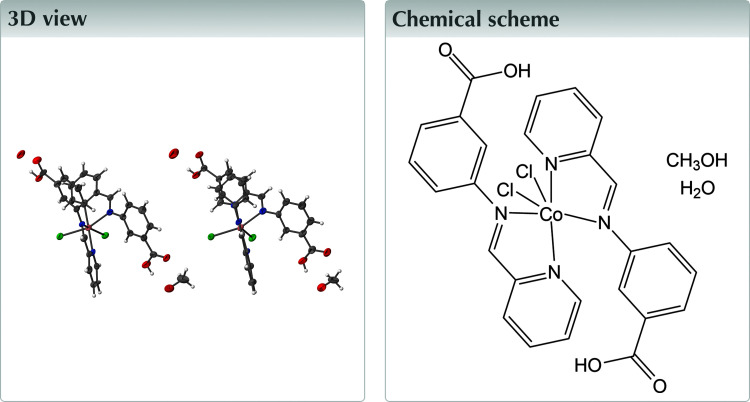

## Structure description

α-Imino­pyridine Schiff bases are electronically analogous to classic bi­pyridines of which metal complexes have shown remarkable photochemical, optical and electrochemical properties (Kitzmann *et al.*, 2023 ▸; Iwai *et al.*, 2024 ▸). α-Imino­pyridines with free carboxyl­ate ends can demonstrate both chelating and bridging functions offering diverse coordination modes (Choudhury *et al.*, 2003 ▸; Buvaylo *et al.*, 2018 ▸). In a continuation of earlier studies on metal complexes with pyridinyl­imino­benzoic acids (Buvaylo *et al.*, 2014 ▸, 2016 ▸, 2018 ▸), we were inter­ested in preparing a cobalt complex with the potentially multidentate 3-{[(pyridin-2-yl)methyl­idene]amino}­benzoic acid (H*L*) ligand that results from the condensation between 3-amino­benzoic acid and 2-pyridine­carbaldehyde. Herein, we report the synthesis and crystal structure of the title complex [Co(H*L*)_2_Cl_2_]·CH_3_OH·H_2_O where the potential bridging function of the carboxyl­ate group did not occur.

**Figure 1:**
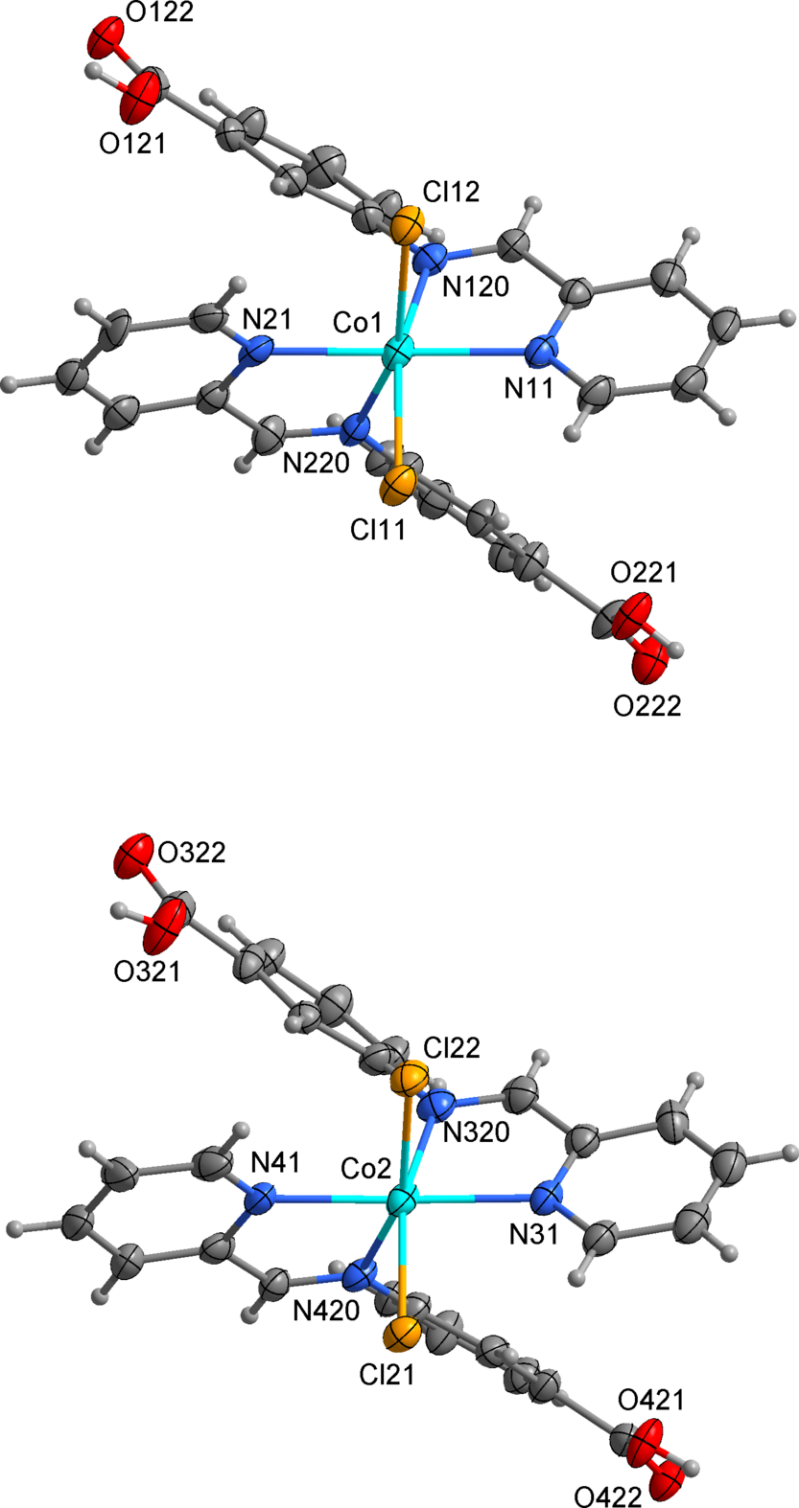
Mol­ecular structure and main atom labelling of [Co(H*L*)_2_Cl_2_]·CH_3_OH·H_2_O, mol­ecules *A* and *B*, with 50% probability displacement ellipsoids. H atoms are shown as spheres of arbitrary radius.

**Figure 2:**
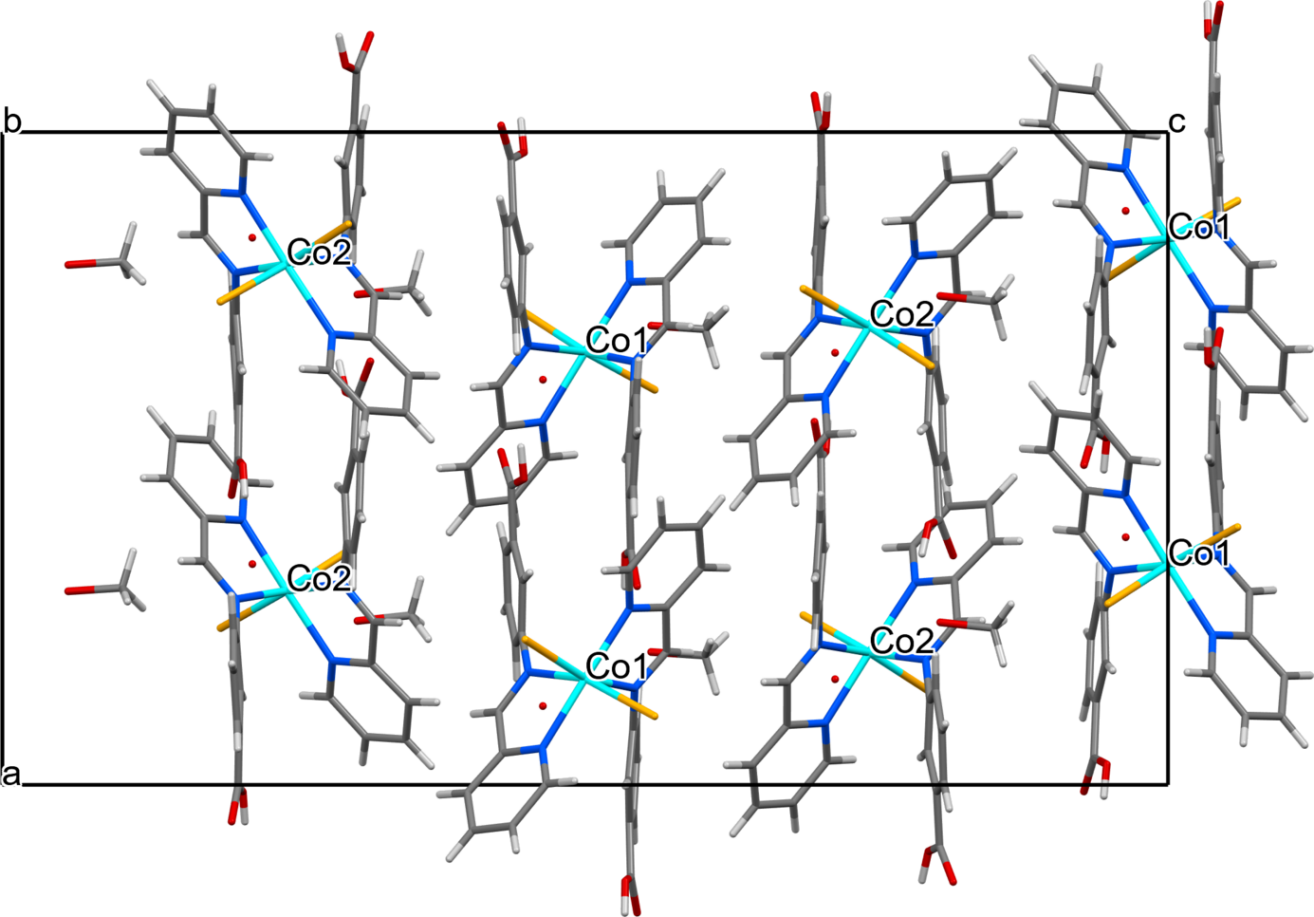
Fragment of the crystal packing of the title compound viewed along the *b* axis.

The compound crystallizes in the ortho­rhom­bic space group *Pna*2_1_ and comprises neutral [Co(H*L*)_2_Cl_2_] mol­ecules as well as solvent mol­ecules of crystallization. In the asymmetric unit, the two independent cobalt complexes (mol­ecules *A* and *B*) adopt similar configurations accommodating two Schiff bases and two *cis*-chlorido ligands in the coordination sphere (Fig. 1 ▸). The metal atoms are distorted octa­hedral, with the Co—N distances lying in the range 2.129 (8)–2.192 (9) Å with the distances to the chloride atoms, 2.409 (3)–2.446 (3) Å, being significantly longer (Table 1 ▸). The *cis* and *trans* angles at the cobalt centres vary in the ranges of 75.3 (3)–107.3 (3) and 158.0 (2)–177.1 (3)°, respectively, support a strong degree of deformation of the metal polyhedra. The C—O bond distances for the incorporated carb­oxy­lic groups [*A*: 1.202 (12), 1.331 (13); 1.181 (11), 1.358 (12) Å; *B*: 1.220 (12), 1.319 (14); 1.207 (12), 1.327 (13) Å] confirm their mol­ecular form. In the crystal, the complex mol­ecules pack loosely with the closest Co⋯Co separation exceeding 7.7 Å (Fig. 2 ▸). The carb­oxy­lic acid groups on the ligands are hydrogen-bonded to the solvent methanol and water mol­ecules (Table 2 ▸). Weak C—H⋯Cl/O inter­molecular inter­actions form a three-dimensional supra­molecular network.

Two monoclinic forms of the free ligand are known belonging to the *C*2/*c* [CSD (Groom *et al.*, 2016 ▸) refcode RIRZIC; Wang *et al.*, 2007 ▸] and *P*2_1_/*c* space groups (RIRZIC01; Tzimopoulos *et al.*, 2010 ▸). The most similar structure to that of the title complex is [Cu(H*L*)_2_Cl]_*n*_Cl_*n*_·2*n*H_2_O built of one-dimensional chains of complex cations joined by weak Cu—Cl coordination bonding, chloride anions and solvent water mol­ecules (TIDREG; Buvaylo *et al.*, 2018 ▸).

## Synthesis and crystallization

2-Pyridine­carbaldehyde (0.19 ml, 2 mmol) was refluxed with 3-amino­benzoic acid (0.28 g, 2 mmol) in methanol (20 ml) in a 50 ml flask for 30 min. The resultant yellow solution was left in open air overnight and used as the ligand without further purification. To the methanol solution with partially deposited H*L* (0.45 g, 2 mmol) CoCl_2_·6H_2_O (0.24 g, 1 mmol) was added. The mixture was heated to 50 °C and magnetically stirred for 20 min until total dissolution of the ligand was observed. The resulting dark-red solution was filtered and allowed to stand at room temperature. Orange prisms of the title complex formed over several days. They were collected by filter-suction, washed with dry ^*i*^PrOH and finally dried in air. Yield: 84% (0.53 g). Analysis calculated for C_27_H_26_Cl_2_CoN_4_O_6_ (MW 632.35): C, 51.28; H, 4.14; N, 8.86%. Found: C, 51.43; H, 4.00; N, 8.73%.

The IR spectrum of the compound is dominated by a sharp absorption at 1716 cm^−1^ attributed to C=O stretching of the carb­oxy­lic acid group. A very broad unstructured band observed in the O—H stretching region at 3398 cm^−1^ indicates the presence of alcohol and carb­oxy­lic acid OH functional groups along with solvent and adsorbed H_2_O mol­ecules involved in extensive hydrogen bonding. The aromatic =C—H and alkyl –C—H stretching is evidenced from the presence of several bands above and below 3000 cm^−1^, respectively. The characteristic ν(C=N) absorption of the Schiff base ligand is identified at 1596 cm^−1^.

## Refinement

Crystal data, data collection and structure refinement details are summarized in Table 3 ▸. Methanol and water mol­ecule O—H hydrogen atoms were not located.

## Supplementary Material

Crystal structure: contains datablock(s) I. DOI: 10.1107/S2414314626002154/tk4120sup1.cif

Supporting information file. DOI: 10.1107/S2414314626002154/tk4120sup3.txt

CCDC reference: 2063851

Additional supporting information:  crystallographic information; 3D view; checkCIF report

## Figures and Tables

**Table 1:** Selected geometric parameters (Å, °)

Co1—N21	2.129 (8)	Co2—N41	2.152 (8)
Co1—N11	2.150 (8)	Co2—N420	2.157 (8)
Co1—N120	2.169 (9)	Co2—N31	2.172 (8)
Co1—N220	2.180 (8)	Co2—N320	2.192 (9)
Co1—Cl11	2.410 (3)	Co2—Cl22	2.409 (3)
Co1—Cl12	2.446 (3)	Co2—Cl21	2.429 (3)
			
N21—Co1—N11	177.1 (3)	N41—Co2—N420	76.4 (3)
N21—Co1—N120	105.7 (3)	N41—Co2—N31	176.6 (3)
N11—Co1—N120	75.9 (3)	N420—Co2—N31	107.0 (3)
N21—Co1—N220	76.6 (3)	N41—Co2—N320	106.4 (3)
N11—Co1—N220	106.1 (3)	N420—Co2—N320	80.2 (3)
N120—Co1—N220	79.8 (3)	N31—Co2—N320	75.3 (3)
N21—Co1—Cl11	88.1 (2)	N41—Co2—Cl22	89.4 (2)
N11—Co1—Cl11	90.9 (2)	N420—Co2—Cl22	158.3 (2)
N120—Co1—Cl11	160.5 (2)	N31—Co2—Cl22	87.7 (2)
N220—Co1—Cl11	90.4 (2)	N320—Co2—Cl22	88.4 (2)
N21—Co1—Cl12	90.0 (2)	N41—Co2—Cl21	87.9 (2)
N11—Co1—Cl12	87.7 (2)	N420—Co2—Cl21	89.9 (2)
N120—Co1—Cl12	87.3 (2)	N31—Co2—Cl21	91.2 (2)
N220—Co1—Cl12	158.0 (2)	N320—Co2—Cl21	159.9 (2)
Cl11—Co1—Cl12	106.75 (10)	Cl22—Co2—Cl21	106.15 (10)

**Table 2:** Hydrogen-bond geometry (Å, °)

*D*—H⋯*A*	*D*—H	H⋯*A*	*D*⋯*A*	*D*—H⋯*A*
O121—H121⋯O3	0.84 (3)	1.87 (6)	2.671 (10)	159 (14)
C16—H16⋯Cl11	0.95	2.72	3.315 (12)	122
C220—H220⋯O222^i^	0.95	2.6	3.090 (12)	112
O221—H221⋯O1	0.82 (3)	1.87 (5)	2.655 (9)	157 (11)
C26—H26⋯Cl12	0.95	2.7	3.308 (11)	123
O321—H321⋯O4	0.82 (5)	1.88 (8)	2.631 (10)	152 (16)
C36—H36⋯Cl21	0.95	2.76	3.359 (11)	122
C426—H426⋯O422^ii^	0.95	2.6	3.497 (11)	157
O421—H421⋯O2	0.83 (3)	1.82 (4)	2.637 (10)	168 (10)
C46—H46⋯Cl22	0.95	2.67	3.275 (12)	122

Symmetry codes: (i) 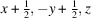; (ii) 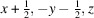.

**Table 3:** Experimental details

Crystal data	
Chemical formula	[CoCl_2_(C_13_H_10_N_2_O_2_)_2_]·CH_4_O·H_2_O
*M*_r_	632.35
Crystal system, space group	Orthorhombic, *Pna*2_1_
Temperature (K)	100
*a*, *b*, *c* (Å)	16.8875 (4), 10.7884 (4), 30.1846 (12)
*V* (Å^3^)	5499.3 (3)
*Z*	8
Radiation type	Cu *K*α
μ (mm^−1^)	7.11
Crystal size (mm)	0.17 × 0.12 × 0.1

Data collection	
Diffractometer	Oxford Diffraction Gemini diffractometer
Absorption correction	Analytical (*CrysAlis PRO*; Rigaku OD, 2015 ▸)
*T*_min_, *T*_max_	0.471, 0.632
No. of measured, independent and observed [*I* > 2σ(*I*)] reflections	29730, 8460, 6837
*R*_int_	0.076
(sin θ/λ)_max_ (Å^−1^)	0.599

Refinement	
*R*[*F*^2^ > 2σ(*F*^2^)], *wR*(*F*^2^), *S*	0.060, 0.161, 1.08
No. of reflections	8460
No. of parameters	739
No. of restraints	5
H-atom treatment	H atoms treated by a mixture of independent and constrained refinement
Δρ_max_, Δρ_min_ (e Å^−3^)	0.67, −0.52
Absolute structure	Flack *x* determined using 2081 quotients [(*I*^+^)−(*I*^−^)]/[(*I*^+^)+(*I*^−^)] (Parsons *et al.*, 2013 ▸)
Absolute structure parameter	0.001 (7)

Computer programs: *CrysAlis PRO* (Rigaku OD, 2015 ▸), *SIR92* (Altomare *et al.*, 1994 ▸), *SHELXL2014* (Sheldrick, 2015 ▸), *Mercury* (Macrae *et al.*, 2020 ▸) and *WinGX* (Farrugia, 2012 ▸).

## full crystallographic data

### Dichloridobis(3-{[(pyridin-2-yl)methylidene]amino}benzoic acid-κ2N,N')cobalt(II) methanol monosolvate monohydrate Crystal data

**Table:**

[CoCl_2_(C_13_H_10_N_2_O_2_)_2_]·CH_4_O·H_2_O	*F*(000) = 2600
*M**_r_* = 632.35	*D*_x_ = 1.528 Mg m^−^^3^
Orthorhombic, *P**n**a*2_1_	Cu *K*α radiation, λ = 1.54178 Å
Hall symbol: P 2c -2n	Cell parameters from 4970 reflections
*a* = 16.8875 (4) Å	θ = 2.9–65.9°
*b* = 10.7884 (4) Å	µ = 7.11 mm^−^^1^
*c* = 30.1846 (12) Å	*T* = 100 K
*V* = 5499.3 (3) Å^3^	Block, orange
*Z* = 8	0.17 × 0.12 × 0.1 mm

### Dichloridobis(3-{[(pyridin-2-yl)methylidene]amino}benzoic acid-κ2N,N')cobalt(II) methanol monosolvate monohydrate Data collection

**Table:**

Oxford Diffraction Gemini diffractometer	8460 independent reflections
Radiation source: sealed X-ray tube	6837 reflections with *I* > 2σ(*I*)
Mirror monochromator	*R*_int_ = 0.076
Detector resolution: 10.4738 pixels mm^-1^	θ_max_ = 67.4°, θ_min_ = 2.9°
ω scans	*h* = −20→14
Absorption correction: analytical (CrysAlisPro; Rigaku OD, 2015)	*k* = −12→12
*T*_min_ = 0.471, *T*_max_ = 0.632	*l* = −36→34
29730 measured reflections	

### Dichloridobis(3-{[(pyridin-2-yl)methylidene]amino}benzoic acid-κ2N,N')cobalt(II) methanol monosolvate monohydrate Refinement

**Table:**

Refinement on *F*^2^	Hydrogen site location: mixed
Least-squares matrix: full	H atoms treated by a mixture of independent and constrained refinement
*R*[*F*^2^ > 2σ(*F*^2^)] = 0.060	*w* = 1/[σ^2^(*F*_o_^2^) + (0.079*P*)^2^ + 6.7183*P*] where *P* = (*F*_o_^2^ + 2*F*_c_^2^)/3
*wR*(*F*^2^) = 0.161	(Δ/σ)_max_ = 0.003
*S* = 1.08	Δρ_max_ = 0.67 e Å^−^^3^
8460 reflections	Δρ_min_ = −0.52 e Å^−^^3^
739 parameters	Absolute structure: Flack *x* determined using 2081 quotients [(*I*^+^)-(*I*^-^)]/[(*I*^+^)+(*I*^-^)] (Parsons *et al.*, 2013)
5 restraints	Absolute structure parameter: 0.001 (7)

### Dichloridobis(3-{[(pyridin-2-yl)methylidene]amino}benzoic acid-κ2N,N')cobalt(II) methanol monosolvate monohydrate Special details

**Table:**

Geometry. All esds (except the esd in the dihedral angle between two l.s. planes) are estimated using the full covariance matrix. The cell esds are taken into account individually in the estimation of esds in distances, angles and torsion angles; correlations between esds in cell parameters are only used when they are defined by crystal symmetry. An approximate (isotropic) treatment of cell esds is used for estimating esds involving l.s. planes.
Refinement. Methanol and water molecule O-H hydrogen atoms were not located.

### Dichloridobis(3-{[(pyridin-2-yl)methylidene]amino}benzoic acid-κ2N,N')cobalt(II) methanol monosolvate monohydrate Fractional atomic coordinates and isotropic or equivalent isotropic displacement parameters (Å^2^)

**Table:**

	*x*	*y*	*z*	*U*_iso_*/*U*_eq_	
Co1	0.33673 (8)	0.60377 (13)	0.50000 (5)	0.0274 (3)	
Co2	0.79734 (8)	0.10038 (14)	0.74368 (5)	0.0293 (4)	
Cl11	0.27741 (13)	0.7474 (2)	0.44862 (9)	0.0363 (5)	
Cl12	0.39479 (12)	0.7277 (2)	0.55959 (8)	0.0339 (5)	
Cl21	0.74015 (13)	0.2333 (2)	0.68717 (8)	0.0348 (5)	
Cl22	0.85857 (13)	0.2367 (3)	0.79683 (8)	0.0387 (6)	
N11	0.2295 (4)	0.5976 (7)	0.5383 (3)	0.0294 (17)	
C12	0.2281 (5)	0.5085 (9)	0.5693 (3)	0.028 (2)	
N120	0.3519 (4)	0.4434 (8)	0.5425 (3)	0.0294 (18)	
C120	0.2959 (5)	0.4248 (9)	0.5699 (3)	0.030 (2)	
H120	0.2982	0.3578	0.5903	0.035*	
C121	0.4158 (5)	0.3579 (9)	0.5411 (3)	0.029 (2)	
C122	0.4923 (5)	0.4028 (9)	0.5374 (3)	0.030 (2)	
H122	0.5015	0.4893	0.5345	0.036*	
C123	0.5566 (5)	0.3192 (9)	0.5380 (3)	0.029 (2)	
C124	0.5424 (6)	0.1939 (10)	0.5413 (4)	0.037 (2)	
H124	0.5857	0.1376	0.542	0.044*	
C125	0.4660 (6)	0.1493 (10)	0.5437 (4)	0.039 (2)	
H125	0.4575	0.0624	0.5453	0.047*	
C126	0.4022 (6)	0.2277 (9)	0.5439 (4)	0.033 (2)	
H126	0.3499	0.1958	0.5459	0.04*	
C127	0.6407 (6)	0.3628 (10)	0.5391 (3)	0.034 (2)	
O121	0.6476 (4)	0.4850 (7)	0.5344 (3)	0.047 (2)	
H121	0.696 (2)	0.502 (12)	0.534 (5)	0.06 (4)*	
O122	0.6957 (4)	0.2936 (7)	0.5446 (3)	0.0382 (17)	
C13	0.1668 (6)	0.4958 (11)	0.6000 (4)	0.039 (2)	
H13	0.1676	0.4322	0.6218	0.047*	
C14	0.1047 (6)	0.5799 (11)	0.5974 (4)	0.042 (3)	
H14	0.0612	0.5734	0.6172	0.051*	
C15	0.1060 (6)	0.6714 (12)	0.5665 (4)	0.042 (3)	
H15	0.0636	0.7292	0.5646	0.051*	
C16	0.1704 (5)	0.6798 (10)	0.5375 (4)	0.039 (2)	
H16	0.1722	0.7457	0.5167	0.047*	
N21	0.4438 (4)	0.6193 (7)	0.4632 (3)	0.0303 (18)	
C22	0.4513 (5)	0.5383 (9)	0.4295 (3)	0.028 (2)	
N220	0.3271 (4)	0.4554 (7)	0.4513 (3)	0.0264 (16)	
C220	0.3880 (5)	0.4480 (10)	0.4257 (4)	0.034 (2)	
H220	0.3914	0.3836	0.4043	0.04*	
C221	0.2670 (5)	0.3656 (9)	0.4455 (3)	0.030 (2)	
C222	0.1872 (5)	0.4064 (9)	0.4464 (3)	0.030 (2)	
H222	0.1752	0.4907	0.4525	0.036*	
C223	0.1276 (5)	0.3237 (9)	0.4384 (3)	0.032 (2)	
C224	0.1445 (6)	0.1985 (9)	0.4323 (4)	0.035 (2)	
H224	0.1026	0.1416	0.427	0.042*	
C225	0.2231 (6)	0.1559 (10)	0.4339 (4)	0.036 (2)	
H225	0.2345	0.0702	0.4305	0.043*	
C226	0.2836 (5)	0.2403 (9)	0.4404 (3)	0.033 (2)	
H226	0.3369	0.2124	0.4415	0.039*	
C227	0.0416 (5)	0.3596 (9)	0.4365 (4)	0.035 (2)	
O221	0.0313 (4)	0.4807 (6)	0.4475 (3)	0.0403 (18)	
H221	−0.017 (2)	0.494 (10)	0.445 (4)	0.04 (3)*	
O222	−0.0101 (4)	0.2898 (7)	0.4279 (3)	0.0420 (18)	
C23	0.5123 (5)	0.5422 (10)	0.3989 (4)	0.036 (2)	
H23	0.515	0.4838	0.3754	0.043*	
C24	0.5698 (5)	0.6347 (9)	0.4036 (3)	0.033 (2)	
H24	0.6132	0.6389	0.3836	0.039*	
C25	0.5628 (5)	0.7194 (10)	0.4374 (4)	0.036 (2)	
H25	0.6001	0.7846	0.4408	0.043*	
C26	0.4984 (6)	0.7065 (10)	0.4669 (3)	0.034 (2)	
H26	0.4942	0.7636	0.4907	0.041*	
N31	0.6901 (4)	0.1115 (7)	0.7832 (3)	0.0279 (17)	
C32	0.6857 (5)	0.0292 (9)	0.8167 (3)	0.032 (2)	
N320	0.8083 (5)	−0.0501 (8)	0.7921 (3)	0.034 (2)	
C320	0.7498 (5)	−0.0608 (9)	0.8192 (4)	0.034 (2)	
H320	0.7486	−0.1259	0.8404	0.041*	
C321	0.8706 (5)	−0.1402 (9)	0.7958 (3)	0.030 (2)	
C322	0.9479 (5)	−0.0959 (9)	0.7940 (3)	0.028 (2)	
H322	0.9583	−0.0111	0.7878	0.034*	
C323	1.0107 (5)	−0.1800 (10)	0.8017 (3)	0.035 (2)	
C324	0.9945 (6)	−0.3051 (10)	0.8074 (4)	0.040 (3)	
H324	1.0367	−0.3618	0.8122	0.049*	
C325	0.9171 (6)	−0.3477 (10)	0.8061 (4)	0.040 (3)	
H325	0.9068	−0.4339	0.8089	0.048*	
C326	0.8550 (6)	−0.2666 (10)	0.8008 (4)	0.036 (2)	
H326	0.8021	−0.2962	0.8005	0.043*	
C327	1.0940 (6)	−0.1358 (10)	0.8040 (4)	0.039 (2)	
O321	1.1020 (4)	−0.0171 (7)	0.7945 (3)	0.053 (2)	
H321	1.146 (4)	0.011 (13)	0.789 (5)	0.09 (5)*	
O322	1.1487 (4)	−0.2020 (7)	0.8157 (3)	0.0455 (19)	
C33	0.6271 (6)	0.0346 (10)	0.8489 (4)	0.038 (2)	
H33	0.6265	−0.0236	0.8725	0.046*	
C34	0.5697 (6)	0.1257 (11)	0.8462 (4)	0.045 (3)	
H34	0.5288	0.1312	0.8677	0.054*	
C35	0.5737 (6)	0.2072 (11)	0.8119 (4)	0.042 (3)	
H35	0.5345	0.2698	0.8091	0.051*	
C36	0.6348 (5)	0.2002 (10)	0.7808 (4)	0.036 (2)	
H36	0.6371	0.2594	0.7576	0.043*	
N41	0.9053 (4)	0.1005 (8)	0.7058 (3)	0.0309 (18)	
C42	0.9086 (5)	0.0137 (9)	0.6732 (3)	0.031 (2)	
N420	0.7843 (4)	−0.0571 (7)	0.7003 (3)	0.0287 (17)	
C420	0.8427 (5)	−0.0734 (9)	0.6733 (3)	0.029 (2)	
H420	0.8428	−0.1421	0.6536	0.035*	
C421	0.7214 (5)	−0.1460 (9)	0.7006 (3)	0.030 (2)	
C422	0.6440 (5)	−0.1016 (10)	0.7038 (3)	0.031 (2)	
H422	0.6336	−0.0154	0.7063	0.038*	
C423	0.5831 (5)	−0.1864 (9)	0.7030 (3)	0.032 (2)	
C424	0.5980 (6)	−0.3130 (10)	0.7013 (4)	0.036 (2)	
H424	0.5552	−0.3701	0.702	0.043*	
C425	0.6749 (6)	−0.3559 (10)	0.6985 (4)	0.037 (3)	
H425	0.6853	−0.4423	0.6969	0.045*	
C426	0.7370 (5)	−0.2716 (10)	0.6983 (3)	0.034 (2)	
H426	0.7901	−0.3002	0.6965	0.041*	
C427	0.4984 (5)	−0.1439 (10)	0.7023 (3)	0.032 (2)	
O421	0.4894 (4)	−0.0222 (7)	0.7063 (3)	0.0406 (18)	
H421	0.444 (3)	0.006 (8)	0.708 (3)	0.03 (3)*	
O422	0.4438 (4)	−0.2143 (7)	0.6967 (3)	0.0377 (17)	
C43	0.9696 (5)	0.0057 (10)	0.6435 (3)	0.034 (2)	
H43	0.9699	−0.0567	0.6213	0.041*	
C44	1.0311 (6)	0.0910 (11)	0.6466 (3)	0.040 (3)	
H44	1.0749	0.0865	0.6269	0.048*	
C45	1.0277 (6)	0.1817 (12)	0.6783 (4)	0.042 (3)	
H45	1.0682	0.2425	0.6804	0.051*	
C46	0.9633 (6)	0.1830 (11)	0.7076 (4)	0.041 (3)	
H46	0.9612	0.2457	0.7296	0.05*	
O1	−0.1207 (4)	0.5239 (7)	0.4637 (3)	0.051 (2)	
O2	0.3382 (5)	0.0347 (9)	0.7144 (4)	0.071 (3)	
O4	1.2511 (4)	0.0472 (8)	0.8046 (3)	0.053 (2)	
C4	1.2597 (8)	0.0352 (13)	0.8502 (4)	0.062 (4)	
H4A	1.2234	0.0923	0.8652	0.092*	
H4B	1.3144	0.0549	0.8586	0.092*	
H4C	1.2475	−0.0502	0.859	0.092*	
O3	0.7965 (4)	0.5400 (7)	0.5566 (3)	0.0509 (19)	
C3	0.8019 (9)	0.5084 (13)	0.6028 (5)	0.069 (4)	
H3A	0.8574	0.5112	0.6122	0.103*	
H3B	0.7809	0.4246	0.6074	0.103*	
H3C	0.7709	0.5677	0.6203	0.103*	

### Dichloridobis(3-{[(pyridin-2-yl)methylidene]amino}benzoic acid-κ2N,N')cobalt(II) methanol monosolvate monohydrate Atomic displacement parameters (Å^2^)

**Table:**

	*U*^11^	*U*^22^	*U*^33^	*U*^12^	*U*^13^	*U*^23^
Co1	0.0189 (6)	0.0283 (8)	0.0352 (8)	0.0013 (6)	0.0000 (6)	−0.0010 (7)
Co2	0.0199 (7)	0.0326 (8)	0.0355 (8)	−0.0009 (6)	−0.0028 (6)	−0.0024 (8)
Cl11	0.0261 (11)	0.0349 (12)	0.0479 (14)	0.0035 (10)	−0.0023 (10)	0.0034 (12)
Cl12	0.0254 (11)	0.0350 (12)	0.0414 (13)	−0.0018 (9)	0.0001 (9)	−0.0055 (11)
Cl21	0.0270 (11)	0.0359 (13)	0.0415 (14)	−0.0013 (9)	−0.0041 (9)	0.0033 (11)
Cl22	0.0289 (12)	0.0468 (15)	0.0403 (14)	−0.0044 (10)	−0.0050 (9)	−0.0097 (12)
N11	0.022 (4)	0.033 (4)	0.033 (4)	0.004 (3)	−0.002 (3)	−0.006 (4)
C12	0.025 (5)	0.033 (5)	0.027 (5)	0.002 (4)	−0.003 (3)	−0.007 (4)
N120	0.025 (4)	0.031 (4)	0.033 (5)	−0.004 (3)	−0.004 (3)	−0.002 (4)
C120	0.023 (4)	0.038 (5)	0.028 (5)	0.001 (4)	−0.001 (4)	0.000 (4)
C121	0.028 (5)	0.033 (5)	0.027 (5)	0.003 (4)	0.003 (4)	0.002 (4)
C122	0.030 (5)	0.032 (5)	0.027 (5)	0.002 (4)	0.002 (4)	0.002 (4)
C123	0.026 (4)	0.037 (5)	0.024 (5)	0.005 (4)	0.003 (4)	−0.001 (4)
C124	0.027 (5)	0.033 (5)	0.051 (6)	0.003 (4)	−0.001 (4)	0.006 (5)
C125	0.040 (6)	0.027 (5)	0.049 (7)	−0.004 (4)	−0.005 (5)	−0.001 (5)
C126	0.026 (5)	0.024 (5)	0.049 (6)	−0.003 (4)	−0.001 (4)	0.002 (5)
C127	0.031 (5)	0.039 (6)	0.031 (5)	−0.006 (4)	0.002 (4)	0.003 (5)
O121	0.026 (4)	0.038 (4)	0.077 (6)	0.001 (3)	0.004 (4)	0.000 (4)
O122	0.022 (3)	0.039 (4)	0.054 (5)	0.006 (3)	−0.002 (3)	0.001 (4)
C13	0.030 (5)	0.052 (7)	0.035 (6)	−0.002 (5)	0.001 (4)	−0.010 (5)
C14	0.021 (5)	0.066 (8)	0.039 (6)	−0.005 (5)	0.000 (4)	−0.014 (6)
C15	0.025 (5)	0.062 (7)	0.040 (6)	0.010 (5)	−0.004 (4)	−0.005 (6)
C16	0.026 (5)	0.043 (6)	0.048 (6)	0.011 (4)	−0.005 (4)	0.003 (5)
N21	0.020 (4)	0.035 (4)	0.036 (5)	0.003 (3)	−0.008 (3)	0.003 (4)
C22	0.022 (4)	0.029 (5)	0.033 (5)	0.004 (4)	−0.004 (4)	0.003 (4)
N220	0.017 (3)	0.028 (4)	0.035 (4)	0.000 (3)	−0.001 (3)	0.004 (4)
C220	0.028 (5)	0.031 (5)	0.041 (6)	0.007 (4)	0.002 (4)	−0.004 (5)
C221	0.021 (4)	0.036 (5)	0.033 (5)	0.001 (4)	−0.004 (4)	0.000 (5)
C222	0.023 (4)	0.029 (5)	0.037 (5)	−0.002 (4)	0.000 (4)	−0.002 (5)
C223	0.022 (4)	0.029 (5)	0.043 (6)	0.001 (4)	0.000 (4)	−0.006 (4)
C224	0.032 (5)	0.030 (5)	0.042 (6)	−0.009 (4)	−0.004 (4)	−0.006 (5)
C225	0.031 (5)	0.036 (6)	0.040 (6)	0.005 (4)	−0.001 (4)	0.001 (5)
C226	0.025 (5)	0.032 (5)	0.041 (6)	0.001 (4)	−0.008 (4)	−0.006 (5)
C227	0.019 (5)	0.034 (5)	0.052 (7)	−0.006 (4)	−0.006 (4)	−0.001 (5)
O221	0.020 (3)	0.030 (4)	0.071 (5)	0.002 (3)	−0.004 (3)	0.005 (4)
O222	0.029 (4)	0.038 (4)	0.059 (5)	−0.003 (3)	0.004 (3)	−0.007 (4)
C23	0.030 (5)	0.038 (6)	0.038 (6)	0.011 (4)	−0.003 (4)	0.003 (5)
C24	0.024 (4)	0.035 (5)	0.039 (6)	0.000 (4)	0.001 (4)	0.008 (5)
C25	0.017 (4)	0.040 (6)	0.051 (6)	−0.011 (4)	0.002 (4)	0.011 (5)
C26	0.030 (5)	0.042 (6)	0.032 (5)	−0.004 (4)	−0.010 (4)	0.012 (5)
N31	0.021 (3)	0.027 (4)	0.036 (5)	0.001 (3)	−0.002 (3)	−0.006 (4)
C32	0.025 (5)	0.037 (5)	0.035 (5)	−0.002 (4)	−0.003 (4)	−0.004 (5)
N320	0.028 (4)	0.037 (5)	0.037 (5)	−0.006 (4)	−0.005 (4)	−0.003 (4)
C320	0.028 (5)	0.035 (5)	0.039 (6)	−0.010 (4)	−0.002 (4)	0.001 (5)
C321	0.027 (5)	0.032 (5)	0.031 (5)	0.004 (4)	−0.005 (4)	0.003 (4)
C322	0.025 (4)	0.031 (5)	0.028 (5)	−0.002 (4)	−0.001 (4)	−0.004 (4)
C323	0.024 (5)	0.040 (6)	0.040 (6)	0.001 (4)	0.000 (4)	−0.016 (5)
C324	0.032 (5)	0.036 (6)	0.053 (7)	0.000 (4)	−0.008 (5)	−0.006 (5)
C325	0.035 (5)	0.034 (6)	0.052 (7)	0.004 (4)	−0.001 (5)	−0.003 (5)
C326	0.027 (5)	0.036 (6)	0.044 (6)	−0.002 (4)	−0.006 (4)	−0.006 (5)
C327	0.030 (5)	0.040 (6)	0.047 (6)	0.005 (5)	−0.001 (4)	−0.007 (5)
O321	0.024 (4)	0.038 (4)	0.096 (7)	−0.001 (3)	0.002 (4)	0.006 (5)
O322	0.029 (4)	0.046 (4)	0.062 (5)	0.009 (3)	−0.003 (3)	0.002 (4)
C33	0.033 (5)	0.042 (6)	0.039 (6)	−0.008 (4)	0.011 (4)	−0.011 (5)
C34	0.030 (5)	0.055 (7)	0.051 (7)	−0.005 (5)	0.005 (5)	−0.010 (6)
C35	0.027 (5)	0.047 (6)	0.053 (7)	0.000 (4)	−0.004 (5)	−0.013 (6)
C36	0.025 (5)	0.040 (6)	0.042 (6)	0.003 (4)	−0.004 (4)	−0.005 (5)
N41	0.019 (3)	0.042 (5)	0.032 (4)	0.000 (3)	−0.003 (3)	0.003 (4)
C42	0.025 (5)	0.036 (5)	0.032 (5)	0.009 (4)	−0.007 (4)	−0.001 (4)
N420	0.022 (4)	0.028 (4)	0.036 (5)	−0.001 (3)	−0.006 (3)	−0.003 (4)
C420	0.022 (4)	0.033 (5)	0.032 (5)	0.005 (4)	−0.002 (4)	−0.001 (4)
C421	0.022 (4)	0.034 (5)	0.034 (5)	−0.002 (4)	−0.004 (4)	−0.004 (4)
C422	0.027 (5)	0.038 (5)	0.029 (5)	0.005 (4)	−0.004 (4)	−0.003 (5)
C423	0.021 (4)	0.040 (6)	0.036 (6)	0.002 (4)	−0.001 (4)	0.006 (5)
C424	0.024 (5)	0.042 (6)	0.042 (6)	−0.006 (4)	−0.004 (4)	−0.005 (5)
C425	0.028 (5)	0.030 (5)	0.054 (7)	0.001 (4)	−0.001 (5)	−0.012 (5)
C426	0.021 (5)	0.043 (6)	0.037 (6)	0.005 (4)	0.002 (4)	0.004 (5)
C427	0.024 (5)	0.043 (6)	0.029 (5)	0.001 (4)	0.001 (4)	0.005 (5)
O421	0.020 (3)	0.034 (4)	0.068 (5)	0.004 (3)	0.003 (3)	−0.003 (4)
O422	0.022 (3)	0.038 (4)	0.053 (5)	−0.004 (3)	−0.001 (3)	−0.003 (4)
C43	0.023 (5)	0.046 (6)	0.034 (6)	0.003 (4)	0.000 (4)	0.001 (5)
C44	0.022 (5)	0.063 (8)	0.035 (6)	−0.006 (5)	−0.001 (4)	0.008 (6)
C45	0.033 (5)	0.065 (7)	0.029 (5)	−0.013 (5)	−0.002 (4)	0.009 (6)
C46	0.033 (5)	0.051 (7)	0.040 (6)	−0.013 (5)	−0.009 (4)	0.008 (5)
O1	0.024 (3)	0.043 (4)	0.085 (6)	0.007 (3)	−0.004 (4)	−0.003 (4)
O2	0.042 (4)	0.059 (6)	0.113 (8)	0.009 (4)	−0.004 (5)	−0.002 (6)
O4	0.040 (4)	0.061 (5)	0.057 (5)	−0.003 (4)	0.001 (3)	−0.001 (4)
C4	0.065 (8)	0.070 (9)	0.050 (8)	−0.035 (7)	0.006 (6)	0.005 (7)
O3	0.046 (4)	0.047 (4)	0.060 (5)	−0.006 (3)	0.007 (4)	−0.003 (4)
C3	0.079 (9)	0.063 (8)	0.064 (9)	−0.024 (7)	0.019 (7)	−0.002 (7)

### Dichloridobis(3-{[(pyridin-2-yl)methylidene]amino}benzoic acid-κ2N,N')cobalt(II) methanol monosolvate monohydrate Geometric parameters (Å, º)

**Table:**

Co1—N21	2.129 (8)	C25—H25	0.95
Co1—N11	2.150 (8)	C26—H26	0.95
Co1—N120	2.169 (9)	N31—C36	1.339 (12)
Co1—N220	2.180 (8)	N31—C32	1.348 (13)
Co1—Cl11	2.410 (3)	C32—C33	1.388 (14)
Co1—Cl12	2.446 (3)	C32—C320	1.456 (14)
Co2—N41	2.152 (8)	N320—C320	1.288 (13)
Co2—N420	2.157 (8)	N320—C321	1.436 (13)
Co2—N31	2.172 (8)	C320—H320	0.95
Co2—N320	2.192 (9)	C321—C322	1.392 (13)
Co2—Cl22	2.409 (3)	C321—C326	1.396 (15)
Co2—Cl21	2.429 (3)	C322—C323	1.414 (13)
N11—C16	1.334 (12)	C322—H322	0.95
N11—C12	1.342 (13)	C323—C324	1.388 (15)
C12—C13	1.396 (14)	C323—C327	1.487 (14)
C12—C120	1.459 (13)	C324—C325	1.386 (14)
N120—C120	1.272 (12)	C324—H324	0.95
N120—C121	1.421 (12)	C325—C326	1.375 (14)
C120—H120	0.95	C325—H325	0.95
C121—C122	1.384 (13)	C326—H326	0.95
C121—C126	1.426 (14)	C327—O322	1.220 (12)
C122—C123	1.412 (13)	C327—O321	1.319 (14)
C122—H122	0.95	O321—H321	0.82 (5)
C123—C124	1.376 (14)	C33—C34	1.383 (16)
C123—C127	1.498 (13)	C33—H33	0.95
C124—C125	1.379 (14)	C34—C35	1.359 (17)
C124—H124	0.95	C34—H34	0.95
C125—C126	1.369 (14)	C35—C36	1.397 (15)
C125—H125	0.95	C35—H35	0.95
C126—H126	0.95	C36—H36	0.95
C127—O122	1.202 (12)	N41—C46	1.324 (13)
C127—O121	1.331 (13)	N41—C42	1.361 (13)
O121—H121	0.84 (3)	C42—C43	1.368 (14)
C13—C14	1.389 (16)	C42—C420	1.456 (14)
C13—H13	0.95	N420—C420	1.291 (12)
C14—C15	1.357 (17)	N420—C421	1.431 (12)
C14—H14	0.95	C420—H420	0.95
C15—C16	1.400 (15)	C421—C426	1.382 (14)
C15—H15	0.95	C421—C422	1.396 (13)
C16—H16	0.95	C422—C423	1.375 (14)
N21—C26	1.323 (13)	C422—H422	0.95
N21—C22	1.346 (13)	C423—C424	1.390 (15)
C22—C23	1.384 (14)	C423—C427	1.503 (13)
C22—C220	1.451 (14)	C424—C425	1.382 (14)
N220—C220	1.289 (12)	C424—H424	0.95
N220—C221	1.414 (12)	C425—C426	1.387 (14)
C220—H220	0.95	C425—H425	0.95
C221—C226	1.389 (14)	C426—H426	0.95
C221—C222	1.418 (12)	C427—O422	1.207 (12)
C222—C223	1.366 (13)	C427—O421	1.327 (13)
C222—H222	0.95	O421—H421	0.83 (3)
C223—C224	1.392 (14)	C43—C44	1.391 (15)
C223—C227	1.506 (13)	C43—H43	0.95
C224—C225	1.405 (14)	C44—C45	1.370 (17)
C224—H224	0.95	C44—H44	0.95
C225—C226	1.383 (14)	C45—C46	1.401 (15)
C225—H225	0.95	C45—H45	0.95
C226—H226	0.95	C46—H46	0.95
C227—O222	1.181 (11)	O4—C4	1.389 (15)
C227—O221	1.358 (12)	C4—H4A	0.98
O221—H221	0.82 (3)	C4—H4B	0.98
C23—C24	1.400 (14)	C4—H4C	0.98
C23—H23	0.95	O3—C3	1.439 (16)
C24—C25	1.375 (15)	C3—H3A	0.98
C24—H24	0.95	C3—H3B	0.98
C25—C26	1.411 (14)	C3—H3C	0.98
			
N21—Co1—N11	177.1 (3)	C25—C24—C23	119.3 (9)
N21—Co1—N120	105.7 (3)	C25—C24—H24	120.4
N11—Co1—N120	75.9 (3)	C23—C24—H24	120.4
N21—Co1—N220	76.6 (3)	C24—C25—C26	118.0 (9)
N11—Co1—N220	106.1 (3)	C24—C25—H25	121
N120—Co1—N220	79.8 (3)	C26—C25—H25	121
N21—Co1—Cl11	88.1 (2)	N21—C26—C25	123.6 (10)
N11—Co1—Cl11	90.9 (2)	N21—C26—H26	118.2
N120—Co1—Cl11	160.5 (2)	C25—C26—H26	118.2
N220—Co1—Cl11	90.4 (2)	C36—N31—C32	118.2 (9)
N21—Co1—Cl12	90.0 (2)	C36—N31—Co2	126.3 (7)
N11—Co1—Cl12	87.7 (2)	C32—N31—Co2	114.9 (6)
N120—Co1—Cl12	87.3 (2)	N31—C32—C33	122.4 (9)
N220—Co1—Cl12	158.0 (2)	N31—C32—C320	116.0 (8)
Cl11—Co1—Cl12	106.75 (10)	C33—C32—C320	121.4 (10)
N41—Co2—N420	76.4 (3)	C320—N320—C321	116.8 (9)
N41—Co2—N31	176.6 (3)	C320—N320—Co2	115.2 (7)
N420—Co2—N31	107.0 (3)	C321—N320—Co2	128.0 (7)
N41—Co2—N320	106.4 (3)	N320—C320—C32	118.5 (10)
N420—Co2—N320	80.2 (3)	N320—C320—H320	120.8
N31—Co2—N320	75.3 (3)	C32—C320—H320	120.8
N41—Co2—Cl22	89.4 (2)	C322—C321—C326	121.1 (9)
N420—Co2—Cl22	158.3 (2)	C322—C321—N320	116.8 (9)
N31—Co2—Cl22	87.7 (2)	C326—C321—N320	122.1 (9)
N320—Co2—Cl22	88.4 (2)	C321—C322—C323	118.4 (9)
N41—Co2—Cl21	87.9 (2)	C321—C322—H322	120.8
N420—Co2—Cl21	89.9 (2)	C323—C322—H322	120.8
N31—Co2—Cl21	91.2 (2)	C324—C323—C322	119.8 (9)
N320—Co2—Cl21	159.9 (2)	C324—C323—C327	119.5 (9)
Cl22—Co2—Cl21	106.15 (10)	C322—C323—C327	120.7 (9)
C16—N11—C12	118.4 (8)	C325—C324—C323	120.3 (10)
C16—N11—Co1	126.8 (7)	C325—C324—H324	119.8
C12—N11—Co1	114.3 (6)	C323—C324—H324	119.8
N11—C12—C13	123.2 (9)	C326—C325—C324	120.7 (10)
N11—C12—C120	116.0 (8)	C326—C325—H325	119.6
C13—C12—C120	120.9 (9)	C324—C325—H325	119.6
C120—N120—C121	118.8 (9)	C325—C326—C321	119.4 (9)
C120—N120—Co1	115.0 (7)	C325—C326—H326	120.3
C121—N120—Co1	126.2 (6)	C321—C326—H326	120.3
N120—C120—C12	118.5 (9)	O322—C327—O321	123.7 (10)
N120—C120—H120	120.7	O322—C327—C323	122.8 (10)
C12—C120—H120	120.7	O321—C327—C323	113.4 (9)
C122—C121—N120	118.9 (9)	C327—O321—H321	119 (10)
C122—C121—C126	120.0 (9)	C34—C33—C32	119.3 (11)
N120—C121—C126	121.1 (8)	C34—C33—H33	120.3
C121—C122—C123	119.5 (9)	C32—C33—H33	120.3
C121—C122—H122	120.3	C35—C34—C33	117.9 (10)
C123—C122—H122	120.3	C35—C34—H34	121
C124—C123—C122	119.7 (9)	C33—C34—H34	121
C124—C123—C127	118.2 (8)	C34—C35—C36	120.9 (10)
C122—C123—C127	121.9 (9)	C34—C35—H35	119.5
C123—C124—C125	120.6 (9)	C36—C35—H35	119.5
C123—C124—H124	119.7	N31—C36—C35	121.2 (11)
C125—C124—H124	119.7	N31—C36—H36	119.4
C126—C125—C124	121.4 (10)	C35—C36—H36	119.4
C126—C125—H125	119.3	C46—N41—C42	117.5 (9)
C124—C125—H125	119.3	C46—N41—Co2	127.3 (8)
C125—C126—C121	118.8 (9)	C42—N41—Co2	114.7 (6)
C125—C126—H126	120.6	N41—C42—C43	123.3 (9)
C121—C126—H126	120.6	N41—C42—C420	114.3 (8)
O122—C127—O121	124.3 (9)	C43—C42—C420	122.4 (9)
O122—C127—C123	122.7 (9)	C420—N420—C421	118.7 (8)
O121—C127—C123	113.0 (9)	C420—N420—Co2	114.3 (6)
C127—O121—H121	107 (9)	C421—N420—Co2	126.8 (6)
C14—C13—C12	117.2 (11)	N420—C420—C42	119.8 (9)
C14—C13—H13	121.4	N420—C420—H420	120.1
C12—C13—H13	121.4	C42—C420—H420	120.1
C15—C14—C13	120.2 (10)	C426—C421—C422	121.2 (9)
C15—C14—H14	119.9	C426—C421—N420	121.0 (8)
C13—C14—H14	119.9	C422—C421—N420	117.8 (9)
C14—C15—C16	119.2 (10)	C423—C422—C421	118.1 (9)
C14—C15—H15	120.4	C423—C422—H422	121
C16—C15—H15	120.4	C421—C422—H422	121
N11—C16—C15	121.8 (11)	C422—C423—C424	121.3 (9)
N11—C16—H16	119.1	C422—C423—C427	120.6 (9)
C15—C16—H16	119.1	C424—C423—C427	118.1 (9)
C26—N21—C22	117.4 (9)	C425—C424—C423	120.1 (9)
C26—N21—Co1	127.2 (7)	C425—C424—H424	120
C22—N21—Co1	115.0 (6)	C423—C424—H424	120
N21—C22—C23	123.7 (9)	C424—C425—C426	119.4 (10)
N21—C22—C220	115.3 (8)	C424—C425—H425	120.3
C23—C22—C220	121.0 (9)	C426—C425—H425	120.3
C220—N220—C221	117.1 (9)	C421—C426—C425	119.9 (9)
C220—N220—Co1	113.0 (6)	C421—C426—H426	120
C221—N220—Co1	129.8 (6)	C425—C426—H426	120
N220—C220—C22	119.9 (9)	O422—C427—O421	123.3 (9)
N220—C220—H220	120.1	O422—C427—C423	122.5 (9)
C22—C220—H220	120.1	O421—C427—C423	114.2 (8)
C226—C221—N220	122.4 (8)	C427—O421—H421	119 (7)
C226—C221—C222	119.7 (8)	C42—C43—C44	118.5 (10)
N220—C221—C222	117.8 (8)	C42—C43—H43	120.8
C223—C222—C221	119.6 (9)	C44—C43—H43	120.8
C223—C222—H222	120.2	C45—C44—C43	119.2 (10)
C221—C222—H222	120.2	C45—C44—H44	120.4
C222—C223—C224	120.5 (9)	C43—C44—H44	120.4
C222—C223—C227	123.3 (9)	C44—C45—C46	118.7 (10)
C224—C223—C227	116.2 (8)	C44—C45—H45	120.6
C223—C224—C225	120.4 (9)	C46—C45—H45	120.6
C223—C224—H224	119.8	N41—C46—C45	122.8 (11)
C225—C224—H224	119.8	N41—C46—H46	118.6
C226—C225—C224	119.2 (10)	C45—C46—H46	118.6
C226—C225—H225	120.4	O4—C4—H4A	109.5
C224—C225—H225	120.4	O4—C4—H4B	109.5
C225—C226—C221	120.5 (9)	H4A—C4—H4B	109.5
C225—C226—H226	119.7	O4—C4—H4C	109.5
C221—C226—H226	119.7	H4A—C4—H4C	109.5
O222—C227—O221	124.9 (9)	H4B—C4—H4C	109.5
O222—C227—C223	123.9 (9)	O3—C3—H3A	109.5
O221—C227—C223	111.2 (8)	O3—C3—H3B	109.5
C227—O221—H221	105 (8)	H3A—C3—H3B	109.5
C22—C23—C24	118.1 (10)	O3—C3—H3C	109.5
C22—C23—H23	121	H3A—C3—H3C	109.5
C24—C23—H23	121	H3B—C3—H3C	109.5

### Dichloridobis(3-{[(pyridin-2-yl)methylidene]amino}benzoic acid-κ2N,N')cobalt(II) methanol monosolvate monohydrate Hydrogen-bond geometry (Å, º)

**Table:**

*D*—H···*A*	*D*—H	H···*A*	*D*···*A*	*D*—H···*A*
O121—H121···O3	0.84 (3)	1.87 (6)	2.671 (10)	159 (14)
C16—H16···Cl11	0.95	2.72	3.315 (12)	122
C220—H220···O222^i^	0.95	2.6	3.090 (12)	112
O221—H221···O1	0.82 (3)	1.87 (5)	2.655 (9)	157 (11)
C26—H26···Cl12	0.95	2.7	3.308 (11)	123
O321—H321···O4	0.82 (5)	1.88 (8)	2.631 (10)	152 (16)
C36—H36···Cl21	0.95	2.76	3.359 (11)	122
C426—H426···O422^ii^	0.95	2.6	3.497 (11)	157
O421—H421···O2	0.83 (3)	1.82 (4)	2.637 (10)	168 (10)
C46—H46···Cl22	0.95	2.67	3.275 (12)	122

Symmetry codes: (i) *x*+1/2, −*y*+1/2, *z*; (ii) *x*+1/2, −*y*−1/2, *z*.
